# Exploring Intergroup Peer Exclusion: Validation of the Latency Social-Psychological Developmental Questionnaire (LSPD)

**DOI:** 10.3390/children10030543

**Published:** 2023-03-13

**Authors:** Hanna Fisher-Grafy, Sonya Meyer

**Affiliations:** 1 Department of Education, Talpiot College of Education, Holon 58500, Israel; 2 Department of Occupational Therapy, Ariel University, Ariel 40700, Israel; sonyam@ariel.ac.il

**Keywords:** intergroup peer exclusion, social rejection, social-psychological normative development, elementary school, latency period, questionnaire

## Abstract

Intergroup peer exclusion has been studied mainly from a pathological aspect. Currently, methods of diagnosis and treatment focus on this pathological point of view. Qualitative research has revealed that social intergroup peer exclusion has a role in the developmental task of the latency stage. The study’s main aim was to develop and validate a quick and easy quantitative questionnaire for use in a school setting that reflects the social developmental variables exposed in previous qualitative research. The 32-item Latency Social-Psychological Developmental questionnaire (LSPD) and the Loneliness and Social Dissatisfaction Questionnaire were administered to 20 Grade 4 and Grade 5 classes at four co-ed public elementary schools (*N* = 373 participants). Factor analysis revealed six developmental factors, and correlations were found between these factors and loneliness. The LSPD is a tool for assessing latency stage development among children who experienced exclusion as well as the developmental status of the entire class. The LSPD can assist in identifying specific development areas to focus on in treatment and intervention.

## 1. Introduction

The term intergroup peer exclusion (sometimes referred to as social rejection) in childhood is a common phenomenon in the latency developmental stage, which includes middle childhood (9 to 11 years of age) [1,2]. Intergroup peer exclusion is more prominent during this developmental stage than earlier or later stages, and focuses on the group level rather than interpersonal friendships [2,3,4,5]. Intergroup peer exclusion encompasses verbal or physical aggression, bullying, isolation, denial of access, and information, humiliation, and shaming in social networks [6]. Generally, it is seen as an inability to form or maintain social relationships [7], and experiences vary in severity from single to chronic exclusion events [8,9]. Whether the exclusion is acute or chronic in nature, children who are excluded, as well as the perpetrators and witnesses of the exclusion, may suffer extreme distress and isolation, with potentially detrimental effects on their current and future health, behavior, learning ability, and emotions [10,11,12,13,14,15].

Arousing widespread concern among educators, the phenomenon has been extensively studied in two main pathology-oriented perspectives for latency-age children. One approach focuses on the personality traits and psychopathology of the children who are rejected that increase their likelihood of becoming objects of intergroup peer exclusion [16,17]. The other approach focuses on the destructive group dynamics of the children who do the rejecting [18,19].

However, almost no studies examined intergroup peer exclusion in a general developmental context or particularly in the context of the unique latency development stage. For example, the social reasoning developmental model focuses on reasoning, judgments, and decisions about moral and social issues and how these processes change across development. According to this approach, interventions need to focus on changing group norms, biases, and prejudices, along with enabling children to understand different sources of inequalities [2,20,21]. The social reasoning developmental model, like other approaches, starts from the premise that intergroup peer exclusion is a challenge that is not necessarily related to the developmental task normatively occurring in the latency phase.

A recent qualitative study sought to examine a normative developmental aspect of this phenomenon, expand knowledge and understanding of development, and add a new dimension to the field of intergroup peer exclusion research. According to this approach, there is significant and unique development during the latency stage. Latency stage development includes several social developmental factors, such as aspiring for group independence and complying more with class social norms and uniformity and less with adult morality. The group flows with decisions that other children made for the class’s actions, plans, and desires. There is acceptance and reconciliation with the existence of a social hierarchy and a reduced egocentric position [22].

Furthermore, the research suggests the possibility of intergroup peer exclusion as a mechanism protecting the group’s integrity; its ability to exist and develop is a top group priority. When the group detects a threat to this development, they activate exclusion [22]. These findings have significant educational and therapeutic implications for prevention and intervention within the school systems.

Based on these qualitative results, a quantitative questionnaire that can measure these developmental aspects is needed. Several available questionnaires are used to diagnose intergroup peer exclusion. Some focus on elderly populations (e.g., [23,24,25]) or are sociometric questionnaires [26,27]. Some self-report questionnaires that detect bullying, social exclusion, and ostracism target younger ages (e.g., [28,29]). However, the focus of the existing questionnaires is to detect the existence of intergroup peer exclusion according to various signs and components and locate the victim, type of injury, and effect on the victim [30]. These effects come in multiple forms; for example, threats or harm that are physical, verbal, social-relational [31,32,33], cyber [29], property, sexual, or racially motivated [34]. Available questionnaires also examine the degree of suffering the victim experiences [35] or the boycott components, the intent to harm, repetition, and power imbalance [36]. These questionnaires are not intended to target the cause of intergroup peer exclusion or guide how it should be addressed. Their goal is to locate and treat the victim according to various approaches.

Intergroup peer exclusion can be identified from various perspectives [37,38]. The perspectives may be those of outsiders’ (e.g., teachers’ [39] or other children’s [39,40,41]), or a self-perspective from the children experiencing the event [37,41,42,43]. We developed this study’s questionnaire as a self-report. When teachers or other children assess the class’s sociometric position, they tend to describe their own perspectives of the social position of the children in the group. This information differs from the individual children’s self-reported perspectives of themselves [40,41]. One child’s experience of exclusion is not necessarily the same as that of the others. Children may perceive themselves as excluded or included, while others perceive the same situation differently [39]. Obtaining these self-reports is necessary to provide appropriate intervention for children who feel excluded [40,44,45].

To the best of our knowledge, no questionnaire diagnoses intergroup peer exclusion according to social psychology or examines the developmental dimensions of the latency stage for all children in a class. However, such a questionnaire might obtain a general picture of the development of the entire class and, within it, the development of the individual. It could identify not only the social exclusion but also the development area where treatment and intervention should be focused.

The newly developed Latency Social-Psychological Developmental questionnaire (LSPD) focuses on the extent to which children comply with social status, class social norms, and social uniformity; independence from adults and adult morality; “group flow;” egocentric position; acceptance; and reconciliation with the existence of a social hierarchy. These factors generate development at the latency age, and delays in their development may lead to intergroup peer exclusion. The LSPD offers an easy, simple-to-administer, in-classroom tool that can pinpoint the social developmental factor that leads some children in the class to exclude other children. It can be beneficial by directing the appropriate intervention to prevent peer exclusion and strengthen inclusiveness.

Therefore, the aims of this study were to (1) develop a quick and easy quantitative questionnaire for use in a school setting that reflects the social developmental variables found in qualitative research, (2) identify delays in the children’s social-psychology development at the individual and class levels, (3) examine the questionnaire’s internal reliability and validity, and (4) explore correlations with feelings of loneliness and social dissatisfaction among children in Grades 4 and 5.

## 2. Materials and Methods

### 2.1. Construction of the LSPD and Item Selection

The LSPD was originally developed in Hebrew and underwent a process of valid translation and back-translation to English (Table 1).

The questionnaire’s content was founded on several resources that established the tool’s content validity: (a) analyses of previously conducted focus groups [22], (b) psychological developmental approaches of the latency stage, (c) moral development of the latency stage, and (d) the social reasoning development approach. Consequently, 36 items were constructed. To establish content and face validity, a panel of four experienced professionals (educational psychologist, educational counselor, art therapist, and movement therapist) conducted an expert review of the selected items. Following this consultation, 32 items were chosen to be included in the questionnaire. Children are asked to score how correct they find each statement, from 1 (not at all correct) to 5 (absolutely correct). A higher score represents better latency social-psychological development. Examples include Item 14, “I behave like everyone else” and Item 30, “I’m friends with the popular kids in my class.” Eight items (Items 5, 7, 18, 19, 25–28) require reverse coding.

### 2.2. Procedure

The final 32-item questionnaire was administered to 20 classes in four co-ed public elementary schools in central Israel as part of a project in collaboration with the Ministry of Education. The study was conducted according to the Declaration of Helsinki guidelines and approved by the Ministry of Education Ethics Committee (7 August 2022, #12618). All parents provided consent in line with the ethics approval, and children completed questionnaires in class independently or with minimal verbal assistance from the project moderators, all qualified educational psychologists.

### 2.3. Participants

A minimum sample size of 210 participants was determined using G*Power software guidelines, considering the medium effect size of 0.5, power = 0.95, and alpha = 0.05 [46]. The inclusion criteria were elementary school pupils in Grades 4 and 5. A total of 373 boys (*n* = 186) and girls (*n* = 187) participated. They were all students in Grade 4 (*n* = 181) or Grade 5 (*n* = 192) in four elementary schools (School A: *n* = 110, B: *n* = 145, C: *n* = 85, D: *n* = 33). The children were aged 10 to 12 years, born in 2012 (45%), 2011 (49.6%), and 2010 (5.4%).

### 2.4. Measures

#### 2.4.1. Demographic Questionnaire

The children completed questions about basic background information, including gender, age, and grade.

#### 2.4.2. Loneliness and Social Dissatisfaction Questionnaire, Modified

The Loneliness and Social Dissatisfaction Questionnaire [47] is a validated 16-item questionnaire that measures feelings of loneliness and social dissatisfaction among school children. As with the original scale, the modified 24-item questionnaire was administered to third- through sixth-grade students. The 16 primary items focus on children’s feelings of loneliness (e.g., “I’m lonely at school”), feelings of social adequacy versus inadequacy (e.g., “I’m good at working with other children at school”), and subjective estimations of peer status (e.g., “I have lots of friends in my class”). Eight more filler items focus on the children’s hobbies or preferred activities (e.g., “I like to paint and draw”). These eight items were included to help children feel more open and relaxed about indicating their feelings on various topics. Items are rated on a Likert scale from 1 (strongly disagree) to 5 (strongly agree). A higher score represents more loneliness. This scale yielded high internal reliability (α = 0.915).

### 2.5. Data Analysis

Data were analyzed using IBM SPSS (Version 26). Descriptive statistics were used to describe the participants. Cronbach’s alpha was calculated to establish reliability, and a level of α ≥ 0.65 was defined as acceptable. Exploratory factor analysis with varimax rotation was applied to the item responses to assist in developing, refining, and evaluating the LSPD. The variables were tested for normality using Shapiro-Wilk tests. We conducted Spearman correlations to assess associations between study variables and Mann-Whitney procedures to compare between groups (e.g., gender). *p*-values lower than 5% are considered significant.

## 3. Results

### 3.1. Construct Validity

An exploratory factor analysis was performed to assess the LSPD construct validity using the criteria of varimax rotation producing orthogonal (independent) factors that explain unique variances and loading of item responses above 0.4. The results revealed six factors composed from 29 items, yielding a cumulative explained variance percentage of 48.2%. See Table 2 for the detailed findings of the factor analysis.

The first factor, social status, includes six items and accounted for 11.26% of the variance measuring social status (e.g., “I’m among the most popular kids in my class”). The second factor, hierarchy acceptance, includes four items and accounted for 8.62% of the variance measuring hierarchy acceptance (e.g., “My class has popular kids”). The third factor, egocentrism, includes four items and accounted for 8.52% of the variance, measuring egocentrism (e.g., “It’s hard for me when other kids decide things in my class and I don’t”). The fourth factor, social norms and uniformity, includes five items and accounted for 6.84% of the variance (e.g., “I behave like everyone else”). The fifth factor, group flow, includes five items and accounted for 6.84% of the variance (e.g., “I don’t like insisting on my own wishes against the wishes of the class”). Finally, the sixth factor, social independence, includes four items and accounted for 5.33% of the variance (e.g., “If kids hurt me, I don’t ask my teacher to deal with them”).

The main study variables were tested for normality distribution by Shapiro-Wilk procedures. These procedures yielded significant results (*p* < 0.001), meaning the variables’ distributions are not proximately normal.

### 3.2. Descriptive Statistics and Correlations

Table 3 presents the descriptive statistics of the main study variables, LSPD scores, and loneliness score [47].

Results showed that high social status (i.e., being well liked and accepted) was correlated with high social norms and uniformity, high tendency to group flow, and low levels of loneliness. In addition, high hierarchy acceptance was positively correlated with egocentrism and loneliness. Egocentrism was negatively correlated with the tendency to group flow and correlated with loneliness. Group flow was negatively correlated with egocentrism and loneliness but positively correlated with social status, social norms, and uniformity. The more the children were connected to the group, had high social status, and could move flexibly with the group and social norms, the lower their scores for egocentrism and loneliness. In addition, egocentrism positively correlated with the acceptance of social hierarchy. That is, children in an egocentric position saw themselves at the center and top of the social ladder and clearly noticed the social hierarchy that eliminated their centrality. Finally, the LSPD total score was negatively associated with loneliness.

### 3.3. Associations between Demographic Information and Study Variables

Table 4 presents the comparison between boys and girls in the study variables. The comparisons were conducted using Mann-Whitney procedures due to the nonnormal distribution of the variables. Results showed that boys scored higher than girls in social status. In addition, the girls reported higher loneliness than the boys.

Table 5 presents the comparison between Grade 4 and Grade 5 in the study variables. The comparisons were conducted using Mann-Whitney procedures due to the nonnormal distribution of the variables.

The results showed that students in Grade 5 scored higher in hierarchy acceptance than students in Grade 4. No other differences were found.

## 4. Discussion

Intergroup peer exclusion, a common phenomenon in the latency developmental stage, seriously harms children in all phases of life, both present and future. The theoretical and practical approaches relate to this phenomenon from a pathological aspect. Accordingly, sociometric questionnaires locate the victims of exclusion. A recent qualitative study examining intergroup peer exclusion from a developmental aspect showed that the focus of the problem with exclusion in latency was not a pathology stemming from the victim’s shortcomings or destructive class dynamics. Instead, intergroup peer exclusion stems from a disturbance in achieving the developmental task in the latency stage [22]. Developmental tasks in latency include the developmental factors of social status, hierarchy acceptance, egocentrism, social norms and uniformity, group flow, and social independence.

So far, no developed questionnaire examines intergroup peer exclusion as a developmental aspect. Thus, the LSPD was constructed to test six developmental factors and was applied in Grades 4 and 5 in Israel. This questionnaire is effective in locating the social status of all children in a class. At the same time, it adds and specifies the developmental area of the class as a whole and of the individual children in it, identifying weaknesses to be strengthened. In this study, we tested the validity and reliability of the quantitative LSPD for use in schools as a quick and easy tool to assess and identify areas for intervention.

Factor analysis revealed the six developmental factors. The first, social status, represents the self-position of the respondent regarding their social status in the class. The literature supports this terminology, defining two dimensions of high social status in childhood: preference (e.g., being well liked and accepted) and popularity (e.g., being seen as popular and influential) [48,49,50,51,52]. The six LSPD items for this factor address the child’s self-perception of preference in the class through their own eyes and those of their classmates, and the more preferred and accepted children in the class. These items include a self-description of the social status of the respondent’s friends with whom the child connects in general and during school recess (when children play freely and social connections are expressed). The social status factor was constructed with these six items based on developmental understandings that emerged from the LSPD’s qualitative findings [22] and that determine social status according to social-psychological development indicators. The novelty of this factor is that it assesses the degree of maturity of the children developing during latency; it does not determine a type of pathology.

The second factor, hierarchy acceptance, represents the respondent’s perception of the existence of social hierarchy in the classroom. Classroom hierarchy has been previously studied relative to gender, race, and other social categories that society constructs and reinforces (e.g., nationality, accent, and language) [53,54,55,56]. The four items in this factor address the child’s perception of the existence of social classes. The social hierarchy acceptance factor is a new factor related to intergroup peer exclusion. It is based on the understanding that the perception of the existence of a social hierarchy has developmental importance and relates to other social factors included in the LSPD (egocentrism, social norms and uniformity, group flow, and social independence). The novelty of this factor is that it was not previously examined in the context of development in the latency stage in general or intergroup peer exclusion in latency in particular.

The third factor, egocentricity, represents the respondent’s feelings regarding their central position in the class. The four LSPD items incorporate the respondent’s feelings about the choices and decisions the children in their class make. They compare the classmates’ attitude toward the respondent with their attitude toward other children in the class. The egocentricity factor is found in Piaget’s theory at younger ages when, at the age of latency, an egocentric position decreases [57,58,59,60]. According to the understanding from new advances [22], the degree of maturity in the egocentric position relates to general development in the latency stage and the ability to flow with the group and its social norms.

The fourth factor, social norms and uniformity, represents the respondent’s conduct in social uniformity according to social norms that prevail in the classroom in various areas: behavior, appearance and clothing, speech, classes, humor, and attitude toward studies. The social norms and uniformity factor is based on previous studies’ findings indicating a relationship between conduct in social uniformity according to social norms and social status [61,62]. This factor has not previously been applied in questionnaires to test development in latency or intergroup peer exclusion factors.

The fifth factor, group flow, represents the respondent’s conduct in conflict situations with the group. The individual’s conduct in conflict situations can range from individual insistence and rowing against the flow to voluntarily joining the group flow according to the classroom’s prevailing social norms. Group flow is a relatively new term used in research about work, music, and sport. However, a comprehensive theoretical model describing Group flow has yet to be developed [63]. The different definitions of group flow are based on the premise that in a situation of group flow, a merging of the individual with the group activity occurs. Thus, the boundaries between the individual and activity blur. In such a situation, there is no vigilance toward the outside world and no self-awareness. The individual joins the group naturally—without conflict or peer pressure—and the group provides the individual with a sense of strength [63,64,65]. The LSPD four group flow items represent conflict situations between the individual and the group, where the individual does not merge and flow with the group’s plans, desires, game choices, or work styles. This fifth factor is based on an understanding of development in which the ability to flow with a group relates to social status, social uniform conduct according to social norms, and an egocentric position [22]. The novelty of this factor is that it has not been tested previously at the age of latency in the contexts of development in general or intergroup peer exclusion in particular.

The sixth factor, group independence, represents a position toward independent social conduct. Independence has been extensively researched regarding early development and adolescence [66,67,68,69,70,71,72]. However, research concerning independence development during latency has been limited, and has focused primarily on teachers’ crucial role in the development of children’s independence [73,74]. The four group-independence items represent each child’s perception of their conduct in social matters on their own. This sixth factor is based on understandings that at the age of latency, independence develops in the group [22]. This factor is novel because it views independence development in latency as a group process and not an individual one.

The LSPD quantitative questionnaire’s internal reliability and validity was examined by comparing the LSPD responses to feelings of loneliness and social dissatisfaction among children in Grades 4 and 5 who are at the age for latency development. Its factors were found to correspond with the previous findings with qualitative focus groups [22]. Factor analysis revealed six developmental factors. Social status, uniformity and norms, and group flow correlate with each other: The more children maintain social uniformity, behave according to social norms, and flow with the group, the higher their social status will be. These three factors were found to have a strong relationship with the total LSPD questionnaire score and a negative relationship with the loneliness index.

Group flow, which was found to have a positive relationship with social status and social norms and uniformity, had a negative relationship with egocentrism and loneliness: The more children are connected to the group, have a high social status, and can flow and move flexibly with the group and its social norms, the less egocentric they are, and the lower the loneliness index. In addition, egocentrism positively correlated with acceptance of social hierarchy. That is, children in an egocentric position notice the social hierarchy that eliminates their centrality.

One unexpected finding was revealed when examining the correlations between variables. Although no significant correlation was found between social independence and the other factors, a positive correlation was found between social independence and the LSPD total score, and a negative correlation was found with the loneliness score. A possible explanation for this is that the class teachers were present while we administered the questionnaires. Their message is usually to seek the teachers’ help during social quarrels. School rules and parental guidance at home support their strong and clear message. The participants possibly identified with this message when they answered the questions that tested social independence in dealing with social quarrels and, in the presence of the teachers, would not raise the possibility of independently solving their social problems.

We found no differences between boys and girls or between Grades 4 and 5 in the variables. However, regardless of the variable tested, the statistical analyses showed that boys reported higher scores than girls in the social status factor. This finding appears consistent with the literature on gender differences in the age of latency. At this age, boys tend to show their courage and strength and avoid showing weakness, thus strengthening their popularity [75,76,77,78]. Girls, on the other hand, demonstrate their weakness and report lower social status and higher loneliness than boys. These findings are consistent with previous studies, which found that girls report loneliness more than boys (e.g., [79,80,81,82]).

## 5. Limitations and Future Research

Some limitations of the current study should be considered. The children studied at public regional schools, where the students are allocated from the same city or areas of generally the same socioeconomic level. In addition, the trait-level differences measured were not examined to validate the LSPD. Future research that considers these aspects and implementation of the questionnaire in additional countries, languages, and cultures could further expand the understanding of intergroup peer exclusions.

## 6. Conclusions

The LSPD’s theoretical contribution lies in its confirmation of the assumptions of the qualitative research [22] showing that intergroup peer exclusion is related to the developmental task in the latency age. This differs from existing approaches in the professional literature, which refer to exclusion as a pathological phenomenon arising from the rejected children’s difficulty, disability, or failure, or from destructive group dynamics [16,17,18,19]. In addition, the LSPD offers a new perspective for the assessment focus. It makes possible the measurement of intergroup peer exclusion in the context of the developmental task in latency and development areas in latency not yet studied. The LSPD’s practical contribution to the field is its ability to locate exclusion at various levels and manifestations differently from the approaches and questionnaires that existed previously [26,27,28,29].

The LSPD identifies not only the victim and type of intergroup peer exclusion, but also developmental areas where there is immaturity. Thus, treatments and interventions can be adjusted depending on the diagnosis. The interventions that exist today for intergroup peer exclusion—which is seen as arising from pathological factors—focus on fighting the intergroup peer exclusion of children or on treating those children’s difficulty or disability. Instead, depending on the LSPD questionnaire results, interventions would focus on both the class and individual levels to improve development areas that are at low levels.

## 7. Practical Implications

The LSPD questionnaire can be used to locate the social developmental factor that must be addressed at the individual or class level. At the individual level, the LSPD can detect a weakness in one of the child’s development areas. For example, the teacher can address the area of social norms and social uniformity by referring to both the individual child and the class.

In the first stage, the teacher can give the individual child a general explanation, according to their level of understanding, of the developmental task at that age—the development of group social skills—and the importance of practicing skills to socially belong to the class. The teacher can describe behaviors that often lead to intergroup peer exclusion and explain how these behaviors harm and disturb class society. An example would be when a class decides on the rules for a game, but one child resists (and even fights) these rules and ruins the game for the other children. The teacher can describe the difficulty of acting as a group when everyone pulls in their own direction and can offer practical ways that the child can strengthen their sense of belonging to the group norms. The teacher can offer the child practical ways to discover involvement and keep up-to-date with their classmates’ activities. For example, connecting to social networks might strengthen their knowledge of prevailing norms for class activities, plans, and conversation topics.

Similarly, teachers can help children at the class level. They can ask the children for examples of social norms that exist in their class (“things all the children in the class agree on and do together”) to help a child who has experienced intergroup peer exclusion identify them and offer tools that raise their awareness of when they deviated from social norms (e.g., using a “social stop sign”). The questionnaire can also help map the areas where the entire classroom needs programs and activities to strengthen classroom cohesion, such as when a large number of children score highly in the egocentricity variable. Class programs and activities could be run to develop the children’s ability to accept situations in which others in the class are also important and that children other than themselves should also be considered.

## Figures and Tables

**Table 1:** The Latency Social-Psychological Developmental Questionnaire (LSPD).

Item		1	2	3	4	5
		Absolutely Not	No	Sometimes	Yes	Absolutely
1	I go with the flow and play with kids in my class even when I don’t like or want to play those games.					
2	I’m among the most popular kids in my class.					
3	Kids in my class don’t reject me.					
4	My class has a class king/queen.					
5	I hate it when people don’t listen to me or do what I want.					
6	I treat my studies like the rest of the kids in my class treat their studies.					
7	When I argue with kids in my class, I never ask the teacher for help.					
8	If we’re working in groups and I think my group isn’t working properly, I’ll still stay with the group.					
9	If we’re working in groups and all the kids in the group say we should do something I really don’t want to do, I’ll still join in.					
10	My class has popular kids.					
11	My class has unpopular kids.					
12	I want to have a similar appearance (clothes, shoes, etc.) as everyone else in my class.					
13	I talk like all the kids in my class.					
14	I behave like everyone else.					
15	I go to extracurricular activities like all the kids in my class.					
16	I watch YouTube videos and play computer games like all the boys/girls in my class.					
17	I laugh at the same things as all the kids in my class.					
18	All social matters should be managed by the kids in the class without adult involvement.					
19	I can manage all my social matters in my class alone, without the help of parents or teachers.					
20	If I’m planning something and my whole class is planning something else, I’ll go with the flow of the whole class.					
21	I don’t like insisting on my own wishes against the wishes of my class.					
22	In my class, we listen to the class king/queen more than to other kids in the class.					
23	I listen to the popular kids in my class.					
24	All the kids in my class think I’m popular.					
25	If kids hurt me, I don’t ask my teacher to deal with them.					
26	It’s hard for me when other kids decide things in my class and I don’t.					
27	It’s hard for me when kids don’t listen to me and don’t do what I want.					
28	It’s hard for me that people listen to and do as other kids say.					
29	At break, I prefer to go out and play with kids from my class and not stay in the classroom.					
30	I’m friends with the popular kids in my class.					
31	If someone asked the class king/queen, he/she would say I’m popular.					
32	During the break, I’m with the popular boys/girls.					

**Table 2:** Loadings of items to factors of the Latency Social-Psychological Developmental Questionnaire (LSPD) scale.

Item (Number and Text)	1	2	3	4	5	6
	Social Status	Hierarchy Acceptance	Egocentrism	Social Norms	Group Flow	Social Independence
24. All the kids in my class think I’m popular.	0.788					
2. I’m among the most popular kids in my class.	0.782					
30. I’m friends with the popular kids in my class.	0.771					
32. During the break, I’m with the popular kids.	0.752					
31. If someone asked the class king/queen, he/she would say I’m popular.	0.744					
29. At break, I prefer to go out and play with kids from my class and not stay in the classroom.	0.423					
22. In my class, we listen to the class king/queen more than to other kids in the class.		0.818				
4. My class has a class king/queen.		0.794				
10. My class has popular kids.		0.709				
11. My class has unpopular kids.		0.558				
27. It’s hard for me when kids don’t listen to me and don’t do what I want.			0.833			
26. It’s hard for me when other kids decide things in my class and I don’t.			0.826			
28. It’s hard for me that people listen to and do as other kids say.			0.719			
5. I hate it when people don’t listen to me or do what I want.			0.678			
14. I behave like everyone else.				0.786		
13. I talk like all the kids in my class.				0.720		
17. I laugh at the same things as all the kids in my class.				0.530		
6. I treat my studies like the rest of the kids in my class treat their studies.				0.465		
12. I want to have a similar appearance (clothes, shoes, etc.) as everyone else in my class.				0.439		
15. I go to extracurricular activities like all the kids in my class.				0.432		
9. If we’re working in groups and all the kids in the group say we should do something I really don’t want to do, I’ll still join in.					0.746	
20. If I’m planning something and my whole class is planning something else, I’ll go with the flow of the whole class.					0.667	
21. I don’t like insisting on my own wishes against the wishes of my class.					0.569	
1. I go with the flow and play with kids in my class even when I don’t like or want to play those games.					0.513	
8. If we’re working in groups and I think my group isn’t working properly, I’ll still stay with the group.					0.432	
19. I can manage all my social matters in my class alone, without the help of parents or teachers.						0.582
7. When I argue with kids in my class, I never ask the teacher for help.						0.572
25. If kids hurt me, I don’t ask my teacher to deal with them.						0.564
18. All social matters should be managed by the kids in the class without adult involvement.						0.527
Percentage of variance	11.26%	8.62%	8.52%	8.02%	6.84%	5.33%
Internal consistency (α)	0.83	0.76	0.78	0.63	0.60	0.42

Note: The items are presented according to their loading size.

**Table 3:** Means, standard deviations, and Spearman correlations between study variables.

Variable	M	SD	1	2	3	4	5	6	7
Social status	3.19	1.01							
Hierarchy acceptance	2.70	1.11	0.066						
Egocentrism	2.80	1.01	0.001	0.235 **					
Social norms and social uniformity	2.56	0.77	0.288 **	0.063	0.086				
Group flow	3.41	0.73	0.158 **	0.048	−0.104 *	0.168 **			
Social Independence	2.87	0.76	0.010	0.090	0.024	0.096	0.060		
LSPD Total	2.86	0.54	0.598 **	0.576 **	0.468 **	0.472 **	0.300 **	0.102 *	
Loneliness	31.18	11.59	−0.488 **	0.234 **	258 **	−0.103 *	−0.207 **	−0.100 *	−0.368 **

Note. * *p* < 0.05, ** *p* < 0.01.

**Table 4:** Comparison between boys and girls in study variables.

Variable	Boys (*n* = 186)	Girls (*n* = 187)	Mann-Whitney	*p*
	M (SD)	M (SD)		
Social status	3.29 (1.02)	3.08 (0.98)	2.09	0.036
Hierarchy acceptance	2.59 (1.03)	2.80 (1.17)	1.55	0.120
Egocentrism	2.75 (0.94)	2.84 (1.06)	0.72	0.470
Social norms and social uniformity	2.60 (0.80)	2.52 (0.73)	0.77	0.440
Group flow/pleasing others	3.37 (0.77)	3.44 (0.68)	1.16	0.240
Social Independence	2.94 (0.75)	2.80 (0.76)	1.48	0.140
LSPD Total	2.86 (0.53)	2.87 (0.54)	0.02	0.980
Loneliness	29.35 (10.13)	33.00 (12.64)	2.73	0.006

**Table 5:** Comparison between Grade 4 and Grade 5 in study variables.

Variable	Grade 4 (*n* = 181)	Grade 5 (*n* = 192)	Mann-Whitney	*p*
	M (SD)	M (SD)		
Social status	3.19 (1.02)	3.18 (1.00)	0.103	0.92
Hierarchy acceptance	2.57 (1.08)	2.81 (1.12)	2.120	0.03
Egocentrism	2.89 (1.06)	2.70 (0.94)	1.400	0.16
Social norms and social uniformity	2.62 (0.80)	2.50 (0.73)	1.210	0.22
Group flow/pleasing others	3.40 (0.75)	3.42 (0.71)	0.880	0.37
Social Independence	2.81 (0.79)	2.93 (0.73)	1.540	0.12
LSPD Total	2.85 (0.56)	2.88 (0.52)	1.050	0.29
Loneliness	31.50 (11.98)	30.87 (11.22)		

## Data Availability

The data presented in this study are available on request from the corresponding author. The data are not publicly available due to privacy and ethical reasons.

## References

[1] Cramer P. (2018). The development of defense mechanisms during the latency period. J. Nerv. Ment. Dis..

[2] Killen M., Rutland A. (2022). Promoting fair and just school environments: Developing inclusive youth. Policy Insights Behav. Brain Sci..

[3] Dake J.A., Price J.H., Telljohann S.K. (2003). The nature and extent of bullying at school. J. Sch. Health.

[4] Cooley S., Burkholder A.R., Killen M. (2019). Social inclusion and exclusion in same-race and interracial peer encounters. Dev. Psychol..

[5] Møller S.J., Tenenbaum H.R. (2011). Danish majority children’s reasoning about exclusion based on gender and ethnicity. Child Dev..

[6] Nergaard K. (2018). “The heartbreak of social exclusion:” Young children’s expressions about how they experience rejection from peers in ECEC. Child Care Pract..

[7] Jiang S., Ngai S.S.Y. (2020). Intergroup peer exclusion and multi-domain well-being in Chinese migrant children: Exploring the psychosocial mechanisms of need satisfaction and need frustration. Child Youth Serv. Rev..

[8] Jambon M. (2020). Peer victimization and sympathy development in childhood: The moderating role of emotion regulation. Soc. Dev..

[9] Rambaran J.A., van Duijn M.A.J., Dijkstra J.K., Veenstra R. (2020). Stability and change in student classroom composition and its impact on peer victimization. J. Educ. Psychol..

[10] Benner A.D., Graham S. (2013). The antecedents and consequences of racial/ethnic discrimination during adolescence: Does the source of discrimination matter?. Dev. Psychol..

[11] Eisenberg N., Spinrad T.L., Morris A., Killen M., Smetana J. (2014). Empathy-related responding in children. Handbook of Moral Development.

[12] McDougall P., Hymel S., Vaillancourt T., Mercer L., Leary M. (2001). The consequences of early childhood rejection. Interpersonal Rejection.

[13] Reijntjes A., Thomaes S., Boelen P., van der Schoot M., de Castro B.O., Telch M. (2011). Delighted when approved by others, to pieces when rejected: Children’s social anxiety magnifies the linkage between self- and other-evaluations. J. Child Psychol. Psychiatry.

[14] Reinhard M.A., Dewald-Kaufmann J., Wüstenberg T., Musil R., Barton B.B., Jobst A., Padberg F. (2020). The vicious circle of intergroup peer exclusion and psychopathology: A systematic review of experimental ostracism research in psychiatric disorders. Eur. Arch. Psychiatry Clin. Neurosci..

[15] Flannery D.J., Todres J., Bradshaw C.P., Amar A.F., Graham S., Hatzenbuehler M., Masiello M., Moreno M., Sullivan R., Vaillancourt T. (2016). Bullying prevention: A summary of the report of the National Academies of Sciences, Engineering, and Medicine. Prev. Sci..

[16] Preti C., Richetin D.P., Fontana A. (2020). Cognitive and emotional components of rejection sensitivity: Independent contributions to adolescent self- and interpersonal functioning. Assessment.

[17] Rubin K., Bukowski W., Parker J., Eisenberg N., Damon W., Lerner. R.M. (2006). Peer interactions, relationships, and groups. Handbook of Child Psychology: Social, Emotional, and Personality Development.

[18] Killen M., Rutland A., Yip T. (2016). Equity and justice in developmental science: Discrimination, intergroup peer exclusion, and intergroup attitudes. Child Dev..

[19] Rohlf H., Krahé B., Busching R. (2016). The socializing effect of classroom aggression on the development of aggression and social exclusion: A two-wave multilevel analysis. J. Sch. Psychol..

[20] McGuire L., Rutland A., Manstead A. Group identity and group norms guide children’s and adolescent’s resource allocation. Proceedings of the British Psychology Society Developmental and Social Section Annual Conference.

[21] Rutland A., Killen M. (2017). Fair resource allocation among children and adolescents: The role of group and developmental processes. Child Dev. Perspect..

[22] Fisher-Grafy H., Rinat H. (2023). “You have no place in the world”: Social exclusion as a developmental mechanism in middle childhood–latency. Contemp. Sch. Psychol..

[23] Gustafson D.P., Harrell T.W. (1970). A comparison of role differentiation in several situations. Organ. Behav. Hum. Perform..

[24] Lieberman M., Gauvin L., Bukowski W.M., White D.R. (2001). Interpersonal influence and disordered eating behaviors in adolescent girls: The role of peer modeling, social reinforcement, and body-related teasing. Eat Behav..

[25] Morán V.E., Ola F.O. (2020). Desarrollo y validación de la Escala de Expectativas de Rechazo Social (ERS) [Development and validation of the Social Exclusion Expectations Scale (SRE)]. Rev. Interam. Psicol..

[26] Terry R., Coie J.D. (1991). A comparison of methods for defining sociometric status among children. Dev. Psychol..

[27] Rosenberg L.A., McHenry T.B., Rosenberg A.M. (1962). Sociometric ratings as predictors of academic performance. J. Appl. Psychol..

[28] Kawamoto T., Nittono H., Ura M. (2015). Trait rejection sensitivity is associated with vigilance and defensive response rather than detection of social exclusion cues. Front. Psychol..

[29] Saylor C.F., Nida S.A., Williams K.W., Taylor L.A., Smyth W., Twyman K.A., Macias M.M., Spratt E.G. (2012). Bullying and Ostracism Screening Scales (BOSS): Development and applications. Child Health Care.

[30] Vessey J., Strout T.D., Difazio R.L., Walker A. (2014). Measuring the youth bullying experience: A systematic review of the psychometric properties of available instruments. J. Sch. Health.

[31] Sutton J., Smith P.K. (1999). Bullying as a group process: An adaptation of the participant role approach. Aggress. Behav..

[32] Austin S., Joseph S. (1996). Assessment of bully/victim problems in 8 to 11 year-olds. Br. J. Educ. Psychol..

[33] Pérez-Morán J.C., Rodríguez-Macías J.C. (2022). Factorial invariance and internal structure of the Scale of the Students’ Role in the Cycle of School Violence (ERECVE). Front. Educ..

[34] Mynard H., Joseph S. (2000). Development of the Multidimensional Peer-Victimization Scale. Aggress. Behav..

[35] Bond L., Wolfe S., Tollit M., Butler H., Patton G. (2007). A comparison of the Gatehouse Bullying Scale and the peer relations questionnaire for students in secondary school. J. Sch. Health.

[36] Xie Z., Man W., Liu C., Fu X. (2022). A PRISMA-based systematic review of measurements for school bullying. Adolesc. Res. Rev..

[37] Berger C., Rodkin P.C. (2009). Male and female victims of male bullies: Social status differences by gender and informant source. Sex Roles.

[38] Graham S., Juvonen J. (2002). Ethnicity, peer harassment, and adjustment in middle school: An exploratory study. J. Early Adolesc..

[39] Sureda-Garcia I., Valera-Pozo M., Sanchez-Azanza V., Adrover-Roig D., Aguilar-Mediavilla E. (2021). Associations between self, peer, and teacher reports of victimization and social skills in school in children with language disorders. Front. Psychol..

[40] Bouman T., van der Meulen M., Goossens F.A., Olthof T., Vermande M.M., Aleva E. (2012). APeer and self-reports of victimization and bullying: Their differential association with internalizing problems and social adjustment. J. Sch. Psychol..

[41] Graham S., Juvonen J. (1998). Self-blame and peer victimization in middle school: An attributional analysis. Dev. Psychol..

[42] Juvonen J., Nishina A., Graham S., Juvonen J., Graham S. (2001). Self-views versus peer perceptions of victim status among early adolescents. Peer Harassment in School: The Plight of the Vulnerable and Victimized.

[43] Leff S., Kupersmidt J., Patterson C., Power T. (1999). Factors influencing teacher identification of peer bullies and victims. Sch. Psychol. Rev..

[44] Hankin B.L., Abramson L.Y. (2001). Development of gender differences in depression: An elaborated cognitive vulnerability–transactional stress theory. Psychol. Bull..

[45] Valera-Pozo M., Adrover-Roig D., Pérez-Castelló J.A., Sanchez-Azanza V.A., Aguilar-Mediavilla E. (2020). Behavioral, emotional and school adjustment in adolescents with and without developmental language disorder (DLD) is related to family involvement. Int. J. Environ. Res. Public Health.

[46] Buchner A., Erdfelder E., Faul F. (2013). G*Power: Statistical Power Analyses for Windows and Mac, Version 3.2.

[47] Asher S.R., Wheeler V.A. (1985). Children’s loneliness: A comparison of rejected and neglected peer status. J. Consult. Clin. Psychol..

[48] Cillessen A.H.N., Marks P.E.L., Cillessen A.H.N., Schwartz D., Mayeux L. (2011). Conceptualizing and measuring popularity. Popularity in the Peer System.

[49] Coie J., Dodge K., Copotelli H. (1982). Dimensions and types of social status: A cross-age perspective. Dev. Psychol..

[50] LaFontana K., Cillessen A. (2002). Children’s perceptions of popular and unpopular peers: A multi-method assessment. Dev. Psychol..

[51] Newcomb A., Bukowski W. (1984). A longitudinal study of the utility of social preference and social impact sociometric classification schemes. Child Dev..

[52] van den Berg Y.H., Lansu T.A., Cillessen A.H. (2020). Preference and popularity as distinct forms of status: A meta-analytic review of 20 years of research. J. Adolesc..

[53] Yazdi H., Barner D., Heyman G.D. (2020). Children’s intergroup attitudes: Insights from Iran. Child Dev..

[54] Heck I.A., Shutts K., Kinzler K.D. (2022). Children’s thinking about group-based social hierarchies. Trends Cogn. Sci..

[55] Kinzler K.D., DeJesus J.M. (2012). Northern = smart and Southern = nice: The development of accent attitudes in the United States. Q. J. Exp. Psychol..

[56] Santhanagopalan R., DeJesus J.M., Moorthy R.S., Kinzler K.D. (2021). Nationality cognition in India: Social category information impacts children’s judgments of people and their national identity. Cogn. Dev..

[57] Piaget J. (1965). The Moral Judgment of the Child.

[58] Piaget J. (2000). Commentary on Vygotsky’s criticisms of language and thought of the child and judgement and reasoning in the child. New Ideas Psychol..

[59] Kesselring T., Mueller U. (2011). The concept of egocentrism in the context of Piaget’s theory. New Ideas Psychol..

[60] Rapanta C. (2023). Piaget, Vygotsky and young people’s argumentation: Sociocognitive aspects and challenges of reasoning “together” and “alone”. Learn. Cult. Soc..

[61] Wilks M., Nielsen M. (2018). Children disassociate from antisocial in-group members. J. Exp. Child Psychol..

[62] Roberts S.O., Ho A.K., Gelman S.A. (2019). The role of group norms in evaluating uncommon and negative behaviors. J. Exp. Psychol..

[63] Pels F., Kleinert J., Mennigen F. (2018). Group flow: A scoping review of definitions, theoretical approaches, measures and findings. PLoS ONE.

[64] Csikszentmihalyi M., Seligman M.E.P., Gillham J. (2000). The contribution of flow to Positive Psychology. The Science of Optimism and Hope.

[65] Engeser S., Schiepe-Tiska A., Peifer C., Peifer C., Engeser S. (2021). Historical lines and an overview of current research on flow. Advances in Flow Research.

[66] Dahlquist L.M. (2015). Parenting and independent problem-solving in preschool children with food allergy. J. Pediatr. Psychol..

[67] Erikson E.H. (1950). Childhood and Society.

[68] Hare A.L. (2015). Undermining adolescent autonomy with parents and peers: The enduring implications of psychologically controlling parenting. J. Res. Adolesc..

[69] Howes C., Lee L., Balter L., Tamis-LeMonda C.S., Balter L., Tamis-LeMonda C.S. (2006). Peer relations in young children. Child Psychology: A Handbook of Contemporary Issues.

[70] McElhaney K., Allen J., Stephenson J., Hare A., Lerner R.M., Steinberg L. (2009). Attachment and autonomy during adolescence. Handbook of Adolescent Psychology.

[71] Power T.G. (2000). Play and Exploration in Children and Animals.

[72] Sroufe L.A., Egeland B., Carlson E.A., Collins W.A. (2005). The Development of the Person.

[73] World Health Organization (2007). International Classification of Functioning, Disability and Health: Children & Youth Version: ICF-CY.

[74] Fadlillah M., Wahab R., Ayriza Y. (2020). Understanding the experience of early childhood education teachers in teaching and training student independence at school. Qual. Rep..

[75] Rose A.J., Rudolph K.D. (2006). A review of sex difference in peer relationship processes: Potential trade-offs for the emotional and behavioral development of girls and boys. Psychol. Bull..

[76] Smith R.L., Rose A.J., Schwartz-Mette R.A. (2009). Relational and overt aggression in childhood and adolescence: Clarifying mean-level gender differences and associations with peer acceptance. Soc. Dev..

[77] Rancourt D., Prinstein M.J. (2010). Peer status and victimization as possible reinforcements of adolescent girls’ and boys’ weight-related behaviors and cognitions. J. Ped. Psychol..

[78] Rodkin P.C., Farmer T.W., Pearl R., Van Acker R. (2000). Heterogeneity of popular boys: Antisocial and prosocial configurations. Dev. Psychol..

[79] Archibald F.S., Bartholomew K., Marx R. (1995). Loneliness in early adolescence: A test of the cognitive discrepancy model of loneliness. Pers. Soc. Psychol. Bull..

[80] Galanaki E. (2004). Are children able to distinguish among the concepts of aloneness, loneliness and solitude. Int. J. Behav. Dev..

[81] Heinrich L.M., Gullone E. (2006). The clinical significance of loneliness: A literature review. Clin. Psychol. Rev..

[82] Salo A.E., Junttila N., Vauras M. (2020). Social and emotional loneliness: Longitudinal stability, interdependence, and intergenerational transmission among boys and girls. Fam. Relat..

